# Influence of maternal lipid levels on adverse pregnancy outcomes in women with gestational diabetes mellitus

**DOI:** 10.3389/fendo.2025.1545393

**Published:** 2025-04-08

**Authors:** Ru Zhao, Jun Hu, Yuanqin Li, Xuetao Chen, Qian Wang, Tingting Wu, Weihong Zhou, Yan Bi, Shanmei Shen, Zhijuan Ge

**Affiliations:** ^1^ Department of Endocrinology, Endocrine and Metabolic Disease Medical Center, Nanjing Drum Tower Hospital, Affiliated Hospital of Medical School, Nanjing University, Nanjing, China; ^2^ Branch of National Clinical Research Centre for Metabolic Diseases, Nanjing, China; ^3^ Department of Health Management Centre, Huadong Sanatorium, Wuxi, China; ^4^ Department of Health Management Centre, Nanjing Drum Tower Hospital, The Affiliated Hospital of Nanjing University Medical School, Nanjing, China

**Keywords:** lipids, gestational diabetes mellitus, adverse outcomes, risk factors, pregnancy

## Abstract

**Objective:**

This study aimed to investigate the effect of mid-pregnancy lipid levels on adverse outcomes in women with gestational diabetes mellitus (GDM) under adequate glycemic control. Whether this effect is independent of factors such as blood glucose was also analyzed.

**Methods:**

We retrospectively analyzed 1,001 women with normal glucose tolerance (NGT) and 1,078 women with GDM under adequate glycemic control from 2015 to 2024. Logistic regression analysis was used to explore the relationship between blood lipids and adverse outcomes. Those with GDM were further classified according to their pre-pregnancy body mass index (BMI), gestational weight gain, glycosylated hemoglobin A1c (HbA1c), and fasting blood glucose (FBG). An interaction model between triglyceride (TG) and pre-pregnancy BMI, gestational weight gain, HbA1c, and FBG on adverse outcomes was constructed.

**Results:**

In GDM, high levels of TG were independent risk factors for preeclampsia (OR = 1.51, 95%CI = 1.18–1.93), preterm birth (OR = 1.68, 95%CI = 1.30–2.18), macrosomia (OR = 1.48, 95%CI = 1.14–1.92), postpartum hemorrhage (OR = 1.33, 95%CI = 1.10–1.61), and intrauterine fetal distress (OR = 1.68, 95%CI = 1.13–2.51). Furthermore, TG had a greater impact on GDM women than on NGT women. In addition, in GDM, high levels of TG were independent risk factors for the above adverse outcomes in the subgroups of pre-pregnancy BMI, gestational weight gain, HbA1c, and FBG (interaction *p* > 0.05).

**Conclusions:**

High levels of TG promoted the occurrence of preeclampsia, preterm birth, macrosomia, postpartum hemorrhage, and intrauterine fetal distress in women with GDM. Furthermore, TG had a greater effect on adverse outcomes in GDM than in NGT women.

## 1 Introduction

Gestational diabetes mellitus (GDM), a condition in which carbohydrate intolerance first develops during pregnancy (1), severely affects pregnant women and infants, with a prevalence of 17.5% in China (2). GDM women and infants are at great risk of perinatal and long-term complications (3, 4). In light of this unfavorable condition, effective intervention strategies are urgently needed to prevent adverse outcomes in women with GDM.

The Hyperglycemia and Adverse Pregnancy Outcome study showed that hyperglycemia positively increases the risk of adverse pregnancy outcomes (5). However, some scholars found that, even if blood glucose control during pregnancy is within the normal range, the risk of adverse pregnancy outcomes is still high (6). Indeed, women with GDM often have a combination of other risk factors for adverse pregnancy outcomes, including advanced maternal age, obesity, and a family history of diabetes mellitus (7, 8). Recent evidence has shown that maternal dyslipidemia is associated with adverse pregnancy outcomes such as preterm birth, large for gestational age (LGA), macrosomia, and intrauterine fetal distress (9, 10). An appropriate increase in maternal triglyceride (TG) and total cholesterol (TC), considered as a physiological adaptation, has a positive impact on the maintenance of pregnancy (11, 12). However, as a result of insulin resistance and placental hormones, women with GDM may easily progress to pathological hyperlipidemia compared with women with normal glucose tolerance (NGT) (13), resulting in numerous adverse outcomes.

Previous studies have shown that dyslipidemia is a risk factor for adverse outcomes such as macrosomia, cesarean section, and neonatal admission to the care unit in women with GDM (14, 15). The relationship between TG levels and pregnancy outcomes has also been investigated in GDM women under glycemic control, but mostly in terms of newborn weight and adiposity accumulation (16, 17). The effect of lipids on adverse pregnancy outcomes in women with GDM in relation to blood glucose remains unknown. Considering that the increase in lipid levels in early pregnancy is not evident, and that the study of lipids in late pregnancy has certain time constraints for clinicians to continuously monitor and intervene in a timely manner, in this study, mid-pregnancy lipids were selected as the study indicators (18).

Thus, women with GDM who had good glycemic control were selected to analyze the role of mid-pregnancy lipids in the development of adverse pregnancy outcomes. An interaction model was further constructed to explore whether the effect of lipids on adverse pregnancy outcomes was associated with other factors such as blood glucose.

## 2 Materials and methods

### 2.1 Study participants

A total of 2,079 pregnant women who were routinely examined and who delivered from 2015 to 2024 were retrospectively analyzed. The participants were divided into NGT and GDM according to the oral glucose tolerance test (OGTT). The eligibility criteria were as follows: 1) the diagnosis of GDM was made using the 75-g OGTT results at 24–28 weeks of gestation (fasting glucose ≥5.1 mmol/L and/or 1-h glucose ≥10.0 mmol/L and/or 2-h glucose ≥8.5 mmol/L, as recommended by the International Association of Diabetes and Pregnancy Study Groups) (19); 2) the enrolled women with GDM were those with good glycemic control during pregnancy (HbA1c <6% and self-monitored fasting blood glucose <5.3 mmol/L during late pregnancy) (20); and 3) women with natural conception singleton pregnancy. The exclusion criteria were as follows: 1) use of insulin therapy or hypoglycemic drugs; 2) use of drugs that may interfere with lipid metabolism; 3) women with pre-pregnancy diabetes; 4) overt diabetes during pregnancy; 5) multiple fetuses; 6) women with uterine abnormalities or reproductive tract infections; and 7) those with severe chronic diseases such as cardiovascular disease, abnormal liver and kidney function, infection, and mental illness.

Ultimately, 1,001 women with NGT and 1,078 women with GDM were recruited into the study. The study was performed in accordance with the Declaration of Helsinki and was carried out with the approval of the Institutional Ethics Committee of Drum Tower Hospital affiliated with Nanjing University Medical School (reference no. 2019-284-01).

### 2.2 Data collection

Pregnancy data were collected through pregnancy cards and electronic medical records, which included the demographic characteristics, the laboratory test indicators, the blood glucose and lipid levels, and the adverse outcomes during pregnancy. The lipid levels were recorded during pregnancy weeks 20–24. The HbA1c levels were recorded at 28–34 weeks of pregnancy. The OGTT was performed at 24–28 weeks of pregnancy. Adverse pregnancy outcomes consisted of preeclampsia, preterm birth, macrosomia, low birth weight (LBW), LGA, small for gestational age (SGA), postpartum hemorrhage, intrauterine fetal distress, and neonatal infection.

### 2.3 Definitions

Preeclampsia is characterized by the onset of hypertension (systolic blood pressure of 140 mmHg or higher or diastolic blood pressure of 90 mmHg or higher on two occasions at least 4 h apart) and proteinuria or in the absence of proteinuria but with the end-organ dysfunction after 20 weeks of gestation (21). Preterm birth is defined as delivery at less than 37 weeks’ gestational age (22). Macrosomia is defined as growth weight beyond 4,000 g or 4,500 g (23). LBW refers to a birth weight less than 2,500 g (24). LGA refers to a birth weight equal to or more than the 90th percentile for a given gestational age, while SGA refers to a birth weight equal to or less than the 10th percentile for a given gestational age (25).

### 2.4 Statistical analysis

Continuous variables were summarized as the mean ± SD or median (interquartile range), while categorical variables were expressed as number of cases (percentage). Continuous variables between the two groups were analyzed using Student’s t test or the Mann–Whitney *U* test, while categorical variables were analyzed using Pearson’s chi-squared test or Fisher’s exact test. Multivariate logistic regression analysis was used to estimate the incidence of adverse outcomes using odds ratios (ORs) and 95% confidence intervals (CIs). Moreover, the interaction model was utilized to explore the relationship between the lipid levels and adverse pregnancy outcomes. A *p* < 0.05 was considered statistically significant. IBM SPSS 26.0 and Empower Stats statistical software were used for the data processing and analysis in this study.

## 3 Results

### 3.1 Baseline data of NGT and GDM women

**Table 1** shows that GDM women were older and had higher pre-pregnancy BMI, lower gestational weight gain, and higher rate of family history of diabetes than NGT women (*p* < 0.05). For previous miscarriages, there were no statistical differences between NGT and GDM (*p* > 0.05).

**Table 1:** Baseline data of NGT and GDM women.

	NGT (*n* = 1,001)	GDM (*n* = 1,078)	*p*-value
Maternal age (years)	29 (27–33)	31 (29–35)	<0.001*
Pre-pregnancy BMI (kg/m^2^)	21.10 (19.57–22.86)	22.58 (20.66–24.78)	<0.001*
Gestational weight gain (kg)	14.00 (11.00–18.00)	11.50 (8.50–14.50)	<0.001*
Miscarriages ≥2, *n* (%)	110 (11.0)	152 (14.1)	0.052
Family history of diabetes, *n* (%)	34 (3.4)	116 (10.8)	<0.001*
HbA1c (%)	–	5.2 (5.0–5.5)	–
TG (mmol/L)	1.82 (1.44–2.33)	2.18 (1.74–2.84)	<0.001*
TC (mmol/L)	5.41 (4.87–5.98)	5.52 (4.85–6.20)	0.030*
LDL-C (mmol/L)	2.63 (2.16–3.10)	2.71 (2.25–3.28)	0.001*
HDL-C (mmol/L)	2.00 (1.72–2.30)	1.88 (1.57–2.19)	<0.001*
Cr (μmol/L)	40.00 (36.00–44.00)	40.00 (37.00–44.00)	0.852
ALT (U/L)	16.00 (9.50–25.80)	15.35 (11.10–22.60)	0.723
AST (U/L)	19.80 (14.80–25.40)	18.00 (14.90–22.70)	0.011*
Ca (mmol/L)	2.37 ± 0.11	2.39 ± 0.12	0.062
P (mmol/L)	1.18 (1.09–1.25)	1.20 (1.13–1.29)	0.068
K (mmol/L)	3.96 ± 0.21	3.95 ± 0.22	0.635
Na (mmol/L)	135.70 (134.80–136.90)	136.25 (135.28–137.20)	0.050

NGT, normal glucose tolerance; GDM, gestational diabetes mellitus; BMI, body mass index; HbA1c, glycosylated hemoglobin A1c; Cr, creatinine; ALT, glutamic–pyruvic transaminase; AST, glutamic–oxaloacetic transaminase; TG, triglyceride; TC, total cholesterol; LDL-C, low-density lipoprotein cholesterol; HDL-C, high-density lipoprotein cholesterol; Ca, calcium; P, phosphorus; K, potassium; Na, natrium

*p < 0.05.

Furthermore, compared with NGT women, GDM women had higher levels of TG [2.18 (1.74–2.84) *vs*. 1.82 (1.44–2.33) mmol/L, *p* < 0.001], TC [5.52 (4.85–6.20) *vs*. 5.41 (4.87–5.98) mmol/L, *p* = 0.030], and LDL-C [2.71 (2.25–3.28) *vs*. 2.63 (2.16–3.10) mmol/L, *p* = 0.001] and lower levels of HDL-C [1.88 (1.57–2.19) *vs*. 2.00 (1.72–2.30) mmol/L, *p* < 0.001]. GDM women had lower glutamic–oxaloacetic transaminase (aspartate aminotransferase, AST) levels. There were no statistical differences with respect to glutamic–pyruvic transaminase (alanine aminotransferase, ALT), kidney function, and electrolytes between the two groups.

### 3.2 Baseline data of pregnant women in the groups with and without adverse outcomes

**Table 2** shows the data of NGT and GDM women divided into two groups according to the occurrence of adverse outcomes. With regard to the clinical characteristics of NGT women, there were no statistically significant differences in age, pre-pregnancy BMI, gestational weight gain, and previous miscarriages between the groups with and without adverse outcomes. NGT women with adverse outcomes had a higher rate of a family history of diabetes compared with the group without adverse outcomes. In terms of the lipid levels in mid-pregnancy, NGT with adverse outcomes showed higher TG [1.90 (1.51–2.57) *vs*. 1.80 (1.42–2.24) mmol/L, *p* = 0.004] and lower HDL-C [1.97 (1.67–2.24) *vs*. 2.02 (1.74–2.32) mmol/L, *p* = 0.020] levels. The levels of TC and LDL-C showed no statistically significant differences between the two groups. In addition, NGT with adverse outcomes had higher levels of ALT and AST. The blood glucose values in the OGTT results, the kidney function, and electrolytes were not determined between the two groups in women with NGT.

**Table 2:** Baseline data of pregnant women in the groups with or without adverse outcomes.

	NGT (*n* = 1001)			GDM (*n* = 1078)		
	Without adverse outcomes (*n* = 650)	With adverse outcomes (*n* = 351)	*p*-value for NGT	Without adverse outcomes (*n* = 632)	With adverse outcomes (*n* = 446)	*p*-value for GDM
Maternal age (years)	30 (28–33)	29 (27–33)	0.070	31 (29–35)	31 (29–34)	0.241
Pre-pregnancy BMI (kg/m^2^)	21.10 (19.46–22.84)	21.10 (19.90–23.06)	0.151	22.41 (20.57–24.22)	22.60 (20.70–25.68)	0.048*
Gestational weight gain (kg)	14.20 (11.50–18.00)	14.00 (11.00–18.00)	0.952	11.00 (8.00–14.00)	12.00 (8.75–15.40)	0.004*
Miscarriages ≥2, *n* (%)	67 (10.3)	43 (12.3)	0.341	91 (14.4)	59 (13.3)	0.421
Family history of diabetes, *n* (%)	15 (2.3)	19 (5.4)	0.010*	65 (10.3)	51 (11.4)	0.554
Mid-pregnancy						
TG (mmol/L)	1.80 (1.42–2.24)	1.90 (1.51–2.57)	0.004*	2.13 (1.71–2.71)	2.31 (1.80–2.96)	0.002*
TC (mmol/L)	5.41 (4.88–5.94)	5.41 (4.85–6.07)	0.676	5.52 (4.85–6.13)	5.53 (4.85–6.29)	0.458
LDL-C (mmol/L)	2.61 (2.17–3.07)	2.68 (2.16–3.12)	0.537	2.71 (2.25–3.28)	2.70 (2.23–3.28)	0.635
HDL-C (mmol/L)	2.02 (1.74–2.32)	1.97 (1.67–2.24)	0.020*	1.88 (1.58–2.17)	1.88 (1.56–2.21)	0.887
75g-OGTT						
FBG (mmol/L)	4.24 (4.04–4.47)	4.26 (4.02–4.48)	0.865	4.58 (4.29–5.05)	4.63 (4.35–5.12)	0.047*
1-h BG (mmol/L)	7.30 (6.30–8.10)	7.30 (6.30–8.30)	0.268	9.92 (9.10–10.60)	10.00 (8.90–10.60)	0.491
2-h BG (mmol/L)	6.20 (5.50–7.00)	6.30 (5.50–7.00)	0.654	8.60 (7.90–9.20)	8.60 (7.60–9.20)	0.449
HbA1c (%)	–	–	–	5.20 (5.00–5.40)	5.20 (5.00–5.50)	0.286
ALT (U/L)	15.55 (8.77–23.50)	18.25 (10.45–30.30)	0.005*	15.20 (11.10–22.50)	13.55 (9.15–19.60)	0.062
AST (U/L)	19.05 (14.60–24.40)	20.90 (15.40–27.20)	0.034*	18.00 (14.90–22.60)	17.75 (14.50–22.50)	0.797
Cr (μmol/L)	40.00 (36.00–43.50)	42.50 (36.00–49.75)	0.137	40.00 (37.00–45.00)	40.00 (37.00–44.00)	0.519
Ca (mmol/L)	2.36 ± 0.11	2.41 ± 0.07	0.061	2.38 ± 0.11	2.41 ± 0.12	0.243
P (mmol/L)	1.17 (1.07–1.25)	1.21 (1.10–1.31)	0.148	1.20 (1.12–1.29)	1.18 (1.13–1.28)	0.624
K (mmol/L)	3.95 (3.83–4.09)	3.93 (3.82–4.08)	0.904	3.93 (3.82–4.10)	3.94 (3.83–4.07)	0.862
Na (mmol/L)	135.7 (134.8–136.9)	135.6 (134.6–136.8)	0.476	136.2 (135.3–137.1)	136.3 (135.2–137.2)	0.936

Without adverse outcomes: no adverse outcomes occurred; With adverse outcomes: one or more adverse outcomes occurred. The *p*-value for NGT refers to the statistical data between NGT without adverse outcomes and NGT with adverse outcomes. The *p*-value for GDM refers to the statistical data between GDM without adverse outcomes and GDM with adverse outcomes. The total *p*-value refers to the statistical data between the total NGT and total GDM.

NGT, normal glucose tolerance; GDM, gestational diabetes mellitus; TG, triglyceride; TC, total cholesterol; LDL-C, low-density lipoprotein cholesterol; HDL-C, high-density lipoprotein cholesterol; OGTT, oral glucose tolerance test; FBG, fasting blood glucose; BG, blood glucose; HbA1c, glycosylated hemoglobin A1c; ALT, glutamic–pyruvic transaminase; AST, glutamic–oxaloacetic transaminase; Cr, creatinine; Ca, calcium; P, phosphorus; K, potassium; Na, natrium.

*p < 0.05.

With regard to the clinical characteristics of GDM women, the group with adverse outcomes had higher pre-pregnancy BMI and gestational weight gain than the group without adverse outcomes. The two groups showed no significant differences in age, previous miscarriages, and family history of diabetes. For the lipid levels in mid-pregnancy, GDM with adverse outcomes had higher TG levels [2.31 (1.80–2.96) *vs*. 2.13 (1.71–2.71) mmol/L, *p* = 0.002] compared with GDM without adverse outcomes. Among the GDM women, TC, LDL-C, and HDL-C showed no significant differences in the two groups. GDM with adverse outcomes had higher FBG levels [4.63 (4.35–5.12) *vs*. 4.58 (4.29–5.05) mmol/L, *p* = 0.047] than GDM without adverse outcomes. In GDM, the two groups showed no significant differences in terms of other laboratory indicators.

### 3.3 Association between lipids in mid-pregnancy and the risk of adverse outcomes among NGT and GDM women

To compare the differences in the impact of blood lipids on adverse pregnancy outcomes between NGT and GDM women and to analyze whether the effect of blood lipids on adverse pregnancy outcomes in GDM is associated with blood glucose or other factors, multivariate logistic regression analyses on the lipids in mid-pregnancy and the risk of adverse outcomes were performed. The results are given in **Table 3**. Moreover, three regression models including different covariates were constructed, and the difference between GDM 1 (ORs adjusted for pre-pregnancy BMI and gestational weight gain) and GDM 2 (ORs adjusted for pre-pregnancy BMI, gestational weight gain, and FBG) lies in the adjustment for FBG. The results showed that, compared with that in NGT women, elevated TG levels in GDM women had greater impact on preeclampsia [OR = 1.51 (95% CI = 1.18–1.93) *vs*. OR = 1.31 (95%CI = 0.90–1.90)], preterm birth [OR = 1.68 (95%CI = 1.30–2.18) *vs*. OR = 1.45 (95%CI = 1.02–2.06)], macrosomia [OR = 1.48 (95%CI = 1.14–1.92) *vs*. OR = 1.48 (95%CI = 1.11–1.98)], postpartum hemorrhage [OR = 1.33 (95%CI = 1.10–1.61) *vs*. OR = 0.93 (95%CI = 0.73–1.18)], and intrauterine fetal distress [OR = 1.68 (95%CI = 1.13–2.51) *vs*. OR = 1.58 (95%CI = 0.79–3.19)]. This significant effect still existed after adjusting for FBG.

**Table 3:** Logistic regression analysis of lipids and the risk of adverse outcomes.

Adverse outcomes	TG	TC	LDL-C	HDL-C
	OR (95%CI)	OR (95%CI)	OR (95%CI)	OR (95%CI)
Preeclampsia				
NGT	1.31 (0.90–1.90)	0.71 (0.45–1.09)	0.90 (0.55–1.48)	0.36 (0.14–0.90)*
GDM 1	1.50 (1.17–1.91)*	1.21 (0.93–1.55)	1.14 (0.83–1.58)	0.84 (0.45–1.56)
GDM 2	1.51 (1.18–1.93)*	1.24 (0.96–1.61)	1.18 (0.85–1.63)	0.85 (0.46–1.58)
Preterm birth				
NGT	1.45 (1.02–2.06)*	1.01 (0.64–1.60)	0.85 (0.50–1.43)	0.27 (0.09–0.75)*
GDM 1	1.65 (1.27–2.13)*	1.21 (0.91–1.62)	1.16 (0.81–1.65)	0.53 (0.26–1.09)
GDM 2	1.68 (1.30–2.18)*	1.21 (0.90–1.61)	1.15 (0.80–1.64)	0.51 (0.25–1.06)
Macrosomia				
NGT	1.48 (1.11–1.98)*	1.19 (0.85–1.66)	1.06 (0.72–1.56)	0.50 (0.23–1.04)
GDM 1	1.48 (1.15–1.91)*	0.99 (0.75–1.32)	0.88 (0.61–1.25)	0.83 (0.42–1.60)
GDM 2	1.48 (1.14–1.92)*	1.04 (0.78–1.39)	0.92 (0.64–1.32)	0.84 (0.43–1.64)
LBW				
NGT	1.25 (0.79–1.98)	1.05 (0.60–1.84)	0.81 (0.42–1.55)	0.25 (0.07–0.89)*
GDM 1	1.02 (0.50–2.06)	0.93 (0.46–1.84)	0.77 (0.33–1.80)	0.36 (0.07–1.87)
GDM 2	1.04 (0.51–2.12)	0.91 (0.45–1.82)	0.76 (0.32–1.77)	0.34 (0.06–1.80)
LGA				
NGT	1.66 (1.25–2.20)*	1.38 (0.99–1.91)	1.29 (0.89–1.87)	0.41 (0.19–0.87)*
GDM 1	1.31 (0.95–1.79)	0.89 (0.63–1.27)	0.77 (0.49–1.22)	0.76 (0.34–1.69)
GDM 2	1.33 (0.96–1.83)	0.89 (0.62–1.29)	0.78 (0.49–1.23)	0.74 (0.33–1.67)
SGA				
NGT	1.09 (0.70–1.69)	0.90 (0.55–1.46)	0.77 (0.44–1.34)	0.41 (0.14–1.17)
GDM 1	0.78 (0.40–1.53)	0.97 (0.56–1.67)	0.88 (0.45–1.74)	0.48 (0.13–1.82)
GDM 2	0.78 (0.40–1.52)	0.98 (0.57–1.71)	0.89 (0.45–1.75)	0.49 (0.13–1.87)
Postpartum hemorrhage				
NGT	0.93 (0.73–1.18)	1.10 (0.88–1.37)	1.08 (0.84–1.38)	1.65 (1.02–2.65)*
GDM 1	1.32 (1.09–1.59)*	1.10 (0.91–1.32)	1.01 (0.80–1.27)	1.05 (0.67–1.64)
GDM 2	1.33 (1.10–1.61)*	1.09 (0.90–1.33)	1.01 (0.79–1.27)	1.05 (0.67–1.64)
Intrauterine fetal distress				
NGT	1.58 (0.79–3.19)	0.68 (0.24–1.89)	0.24 (0.06–0.97)*	0.88 (0.09–8.01)
GDM 1	1.66 (1.12–2.47)*	1.09 (0.66–1.78)	1.04 (0.56–1.94)	0.26 (0.07–0.95)*
GDM 2	1.68 (1.13–2.51)*	1.08 (0.66–1.78)	1.04 (0.55–1.93)	0.25 (0.06–0.94)*
Neonatal infection				
NGT	0.11 (0.01–0.93)*	0.82 (0.32–2.10)	1.26 (0.47–3.38)	0.23 (0.03–1.94)
GDM 1	0.95 (0.56–1.60)	1.08 (0.68–1.72)	1.08 (0.60–1.94)	0.58 (0.19–1.81)
GDM 2	0.98 (0.58–1.66)	1.05 (0.65–1.68)	1.05 (0.58–1.90)	0.55 (0.17–1.71)

NGT, Adjusted odds ratios were adjusted for pre-pregnancy BMI, gestational weight gain, family history of diabetes, glutamic–pyruvic transaminase (ALT), and glutamic–oxaloacetic transaminase (AST). GDM 1, Adjusted odds ratios were adjusted for pre-pregnancy BMI and gestational weight gain. GDM 2, Adjusted odds ratios were adjusted for pre-pregnancy BMI, gestational weight gain, and fasting blood glucose (FBG).

TG, triglyceride; TC, total cholesterol; LDL-C, low-density lipoprotein cholesterol; HDL-C, high-density lipoprotein cholesterol; LBW, low birth weight; LGA, large for gestational age; SGA, small for gestational age. *P <0.05.

### 3.4 Pearson’s correlation analysis between TG and various other factors in GDM women

**Figure 1:**
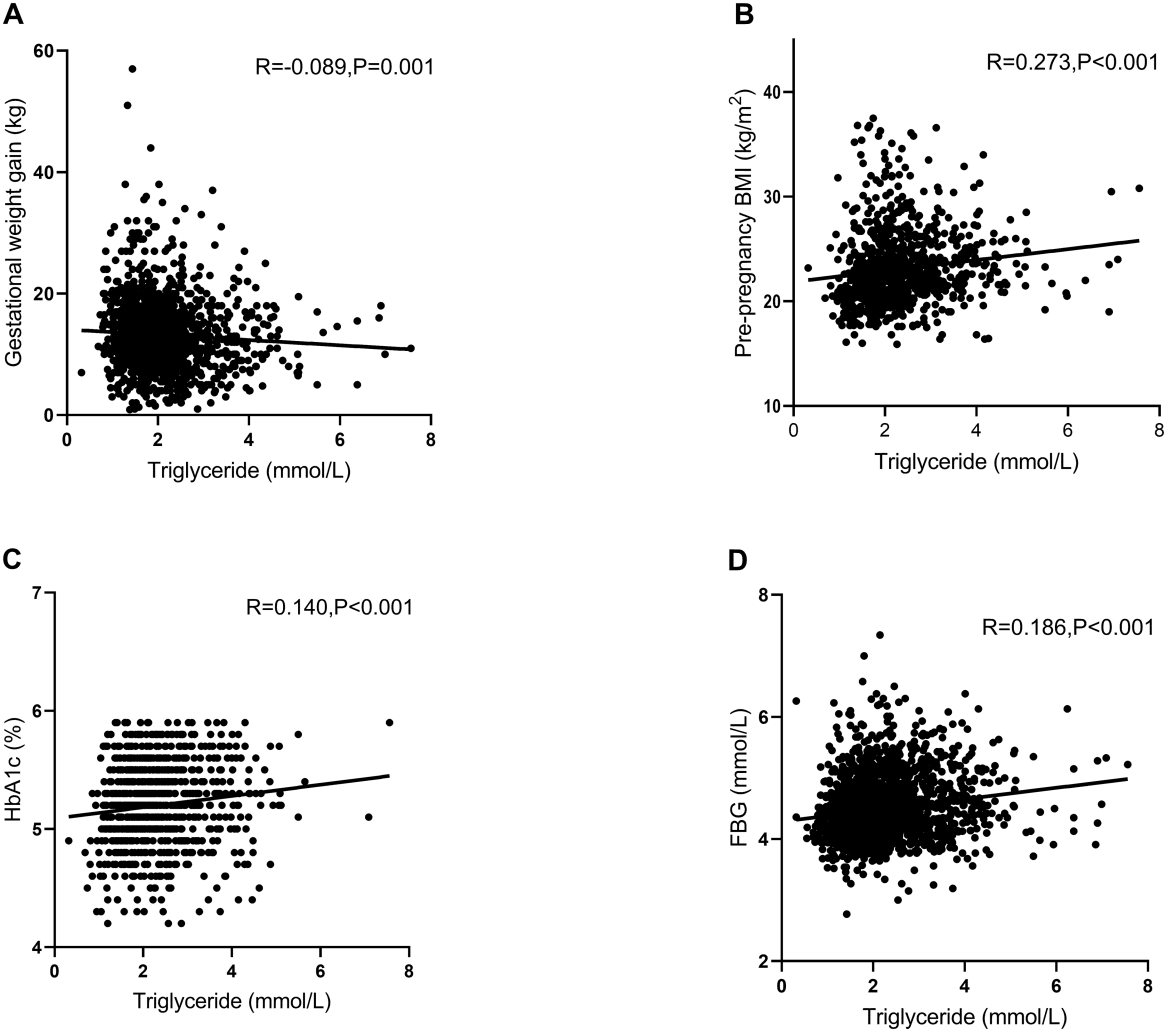
Pearson’s correlation analysis between triglyceride (TG) and various other factors in women with gestational diabetes mellitus (GDM). **(A)** Scatter plot of TG and gestational weight gain. TG was significantly negatively correlated with gestational weight gain (*R* = −0.089, *p* = 0.001). **(B)** Scatter plot of TG and pre-pregnancy BMI. TG was significantly positively correlated with pre-pregnancy BMI (*R* = 0.273, *p* < 0.001). **(C)** Scatter plot of TG and HbA1c. TG was significantly positively correlated with HbA1c (*R* = 0.140, *p* < 0.001). **(D)** Scatter plot of TG and FBG. TG was significantly positively correlated with FBG (*R* = 0.186, *p* < 0.001). R, relevant coefficients; BMI, body mass index; FBG, fasting blood glucose; HbA1c, glycosylated hemoglobin A1c.

The results showed that TG was significantly positively correlated with pre-pregnancy BMI (*R* = 0.273, *p* < 0.001), HbA1c (*R* = 0.140, *p* < 0.001), and FBG (*R* = 0.186, *p* < 0.001) and negatively correlated with gestational weight gain (*R* = −0.089, *p* = 0.001) in GDM women (**Figure 1**).

### 3.5 Interaction analysis between TG and various other factors in GDM women

**Figure 2:**
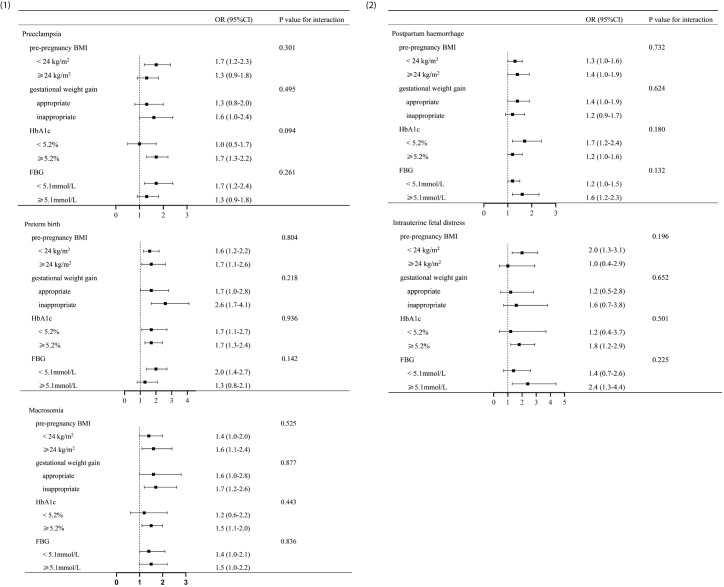
Analysis of the interaction between triglyceride (TG) and various factors in gestational diabetes mellitus (GDM) women. (1, 2) Analysis of the interaction between TG and various other factors in women with GDM. Adjusted odds ratios were adjusted for pre-pregnancy BMI, gestational weight gain, and FBG. The *p*-value for interaction was >0.05. There was no significant distinction in the impact of TG on adverse pregnancy outcomes between individuals with different levels of pre-pregnancy BMI (<24 or ≥24 kg/m^2^), gestational weight gain (appropriate or inappropriate), HbA1c (<5.2% or ≥5.2%), and FBG (<5.1 or ≥5.1 mmol/L). BMI, body mass index; FBG, fasting blood glucose; HbA1c, glycosylated hemoglobin A1c.

**Figure 1** shows that the TG levels were associated with pre-pregnancy BMI, gestational weight gain, HbA1c, and FBG. On this basis, the women with GDM were grouped by pre-pregnancy BMI, gestational weight gain, HbA1c, and FBG (**Figures 2-1, 2-2**). In addition, a model was constructed to investigate the interaction between pre-pregnancy BMI, gestational weight gain, HbA1c, and FBG and the effect of TG on adverse pregnancy outcomes. The results showed that there was no interaction between the effect of TG on adverse pregnancy outcomes and pre-pregnancy BMI, gestational weight gain, HbA1c, and FBG, with the interaction *p*-value >0.05.

## 4 Discussion

In this retrospective study, it was observed that GDM women with good blood glucose control appeared to have higher lipid levels than NGT women. Furthermore, elevated TG levels during mid-pregnancy were positively associated with adverse outcomes. Notably, GDM women experienced a greater effect of the TG levels on adverse outcomes than NGT women. This suggests that, for women with GDM, on the basis of managing blood glucose, attention should also be paid to the blood lipids, particularly to the levels of TG.

There are several factors that cause elevated blood lipids during pregnancy. Due to the special metabolic state during pregnancy, the blood lipid levels of pregnant women show dynamic changes. Compared with those in non-pregnant individuals, the blood lipids levels during pregnancy may increase physiologically (26). The results of this study showed that GDM women had higher levels of TG and TC compared with NGT women, which is consistent with previous studies (27, 28). Yazıcı et al. demonstrated that increased lipid levels and an altered intracellular signaling metabolism are associated with insulin resistance (29). As a result of insulin resistance, the main sources of TG in the liver are abnormally elevated (13). Therefore, pathological hyperlipidemia is more likely to occur among GDM women. Moreover, owing to the widespread application of assisted reproductive practice such as intrauterine insemination and autologous platelet-rich plasma, women undergoing these procedures may face physiological changes (30, 31). Research shows that women who have received assisted reproduction may experience ovarian stimulation and changes in hormone levels. This could exacerbate insulin resistance and cause disturbances in the glucose and lipid metabolism among pregnancies and the offspring (32, 33). Hence, in this study, women who received assisted reproduction were excluded.

Previous studies have shown that dyslipidemia is a risk factor for preterm birth, macrosomia, and preeclampsia (34, 35). Similarly, we found that the TG levels in the group with adverse outcomes were higher than those in the group without adverse outcomes in both NGT and GDM women. A number of scholars have noted that the TG levels in GDM women are associated with macrosomia and preeclampsia and that the effect is more significant than that in NGT women (14). However, the aforementioned study did not take into account the relationship between the impact of lipids on adverse outcomes and the blood glucose factors. The American Diabetes Association (ADA) recommends an ideal HbA1c target of <6.0% in the absence of hypoglycemia (20). In order to investigate whether glycemic factors are involved in the effect of lipids on adverse pregnancy outcomes in women with GDM, the included GDM population all met the glycemic control target. The logistic regression analysis revealed that, after adjustment for glycemic factors (GDM 2), TG remained an independent risk factor for preeclampsia, preterm birth, macrosomia, postpartum hemorrhage, and intrauterine fetal distress. Furthermore, consistent with the study by Shi et al. (14), it was found that, compared with that in NGT women, the effect of TG on the aforementioned adverse outcomes was more pronounced in GDM women. The development of preeclampsia is influenced by numerous factors, and we have considered the effect of pre-pregnancy BMI, gestational weight gain, and blood glucose via the nadir criteria and multifactorial analyses. Currently, there is insufficient evidence to explain the mechanism of preeclampsia caused by TG. Chen et al. (36) established a preeclampsia mouse model using hypoxia-inducible factor 1α and found high levels of blood lipids and urinary protein in the mouse placenta, accompanied by mitochondrial dysfunction. After improvement of mitochondrial function, the blood lipids and urinary protein levels decreased accordingly, hinting that placental mitochondrial dysfunction may be involved in the occurrence and the development of preeclampsia caused by blood lipids. Several studies have indicated that an abnormally elevated TG increases blood viscosity and the risk of thrombosis, leading to inadequate placental perfusion, nutrient transfer disorder, fetal ischemia, and hypoxia. As a result, high levels of TG induce preterm birth and intrauterine fetal distress (37, 38).

A summary of 46 studies revealed that high maternal TG and low HDL-C increased the risk of macrosomia (39). This may be attributed to the point that placental lipase hydrolyzes TG into fatty acids, which in turn leads to the abnormal accumulation of fetal fat and to excessive growth (40, 41). However, an association between HDL-C and macrosomia was not found, which may be due to differences in the distribution of population characteristics.

It was found that TG was associated with gestational weight gain, pre-pregnancy BMI, HbA1c, and FBG. In order to further explore whether the effect of TG on adverse pregnancy outcomes was associated with the above factors, interaction analyses based on the subgroups were performed. There was no interaction observed between TG and gestational weight gain, pre-pregnancy BMI, HbA1c, and FBG (interaction *p*-value >0.05), indicating that the impact of TG on adverse pregnancy outcomes was not affected by the above factors. There was no significant distinction in the impact of TG on adverse pregnancy outcomes between individuals with different levels of pre-pregnancy BMI (<24 or ≥24 kg/m^2^), gestational weight gain (appropriate or inappropriate), HbA1c (<5.2% or ≥5.2%), and FBG (<5.1 or ≥5.1 mmol/L). This further suggests that TG had an independent effect on adverse pregnancy outcomes. Hence, even for individuals with relatively suitable levels of pre-pregnancy BMI, gestational weight gain, HbA1c, and FBG, attention should still be paid to the management of lipids. Indeed, a meta-analysis demonstrated that high levels of TG have a more significant impact on macrosomia in pregnant women who are overweight or obese before pregnancy (39). In this study, the ORs of macrosomia in GDM pregnant women with pre-pregnancy BMI ≥24 kg/m^2^ were higher than those in pregnant women with BMI <24 kg/m^2^; however, there were no statistically significant differences in the interaction between the groups. We speculate that the difference in the results may be related to the limitation in the sample size.

This study has several limitations. Firstly, the blood lipids indicators did not include free fatty acids. Secondly, although we adjusted for blood glucose factors in women with GDM, there are still other unknown confounding factors. Finally, considering that the lipid levels are related to dietary habits and regional distribution and this study only included the population of the Nanjing area, further research encompassing wider areas should be performed.

In summary, this study found that high levels of TG are independent risk factors for preeclampsia, preterm birth, macrosomia, postpartum hemorrhage, and intrauterine fetal distress. In addition, TG had a greater impact on GDM than on NGT women. Clinicians should monitor the blood lipid levels early and dynamically in women with GDM and to recommend timely lifestyle interventions, which can help reduce the incidence of adverse outcomes (42).

## Data Availability

The raw data supporting the conclusions of this article will be made available by the authors, without undue reservation.
